# Bellini Duct Carcinoma Misdiagnosed with Urothelial Papillary Carcinoma

**DOI:** 10.1155/2020/3174674

**Published:** 2020-02-10

**Authors:** Joey El Khoury, Maher Abdessater, Rami Halabi, Fadi Nasr, Johnny Boustany, Anthony Kanbar, Charbel El Hachem, Raghid El Khoury

**Affiliations:** ^1^Faculty of Medicine and Medical Sciences, Holy Spirit University of Kaslik (USEK), Jounieh, Lebanon; ^2^Department of Urology, University Hospital Center-Notre Dame des Secours, Byblos, Lebanon; ^3^Department of Oncology, University Hospital Center-Notre Dame des Secours, Byblos, Lebanon

## Abstract

**Background:**

Collecting (Bellini) duct carcinoma (CDC) or Bellini duct carcinoma (BDC) is a rare subtype of kidney tumors, accounting for less than 3% and known to have the worst prognosis. It is known to have multiple clinical presentations; this is why it can be easily misdiagnosed. The aim of this article is to present a case of CDC that was initially misdiagnosed with urothelial papillary carcinoma (UPC) in a 41-year-old male. *Case Presentation*. Our patient presented with a left flank pain evolving for one month and one episode of gross macroscopic hematuria. Upon presentation, he had left costovertebral angle tenderness. Initial lab tests were normal. Computed tomography revealed a 5 cm solid mass of the left renal pelvis and multiple infracentimetric perihilar lymph nodes. Subsequently, the patient had left nephroureterectomy. Microscopic examination showed the presence of a high-grade urothelial papillary carcinoma of the renal pelvis' lumen. All four of the dissected lymph nodes showed disease metastasis. Three years after establishing the diagnosis, the patient presented again for chronic abdominal pain, with a recent history of weight loss. CT scan showed a left paraaortic mass infiltrating the left psoas muscle over a length of 12 cm. Immunohistochemical profiling of this mass confirmed the diagnosis of Bellini duct carcinoma, rejecting the initial diagnosis of UPC. Therefore, the patient required a cisplatin-gemcitabine-based chemotherapy regimen.

**Conclusion:**

BDC remains one of the rare aggressive subtypes of RCC, having a multitude of initial clinical presentations and an unfavorable prognosis. In this patient, CDC was masquerading as a transitional cell carcinoma that should always be kept in mind as a possible presentation. Corresponding early imaging and histopathology exams are primordial for a correct diagnosis and thus a better prognosis.

## 1. Introduction

Collecting duct carcinoma (CDC) is a rare subtype of kidney tumors, accounting for less than 3% and known to have the worst prognosis with its tendency to early metastasis [1]. CDC is also called Bellini duct carcinoma (BDC) because it concerns the distal medullar segment of the collecting duct of Bellini [2]. Commonly, the disease is diagnosed between ages 40 and 71, with a 2 : 1 male to female ratio [1]. African descents are more touched than Caucasians. Unfortunately, given the large spectrum of initial presentation signs and symptoms that mimics other types of renal carcinomas, CDC can be easily misdiagnosed [3]. We hereby report the case of a middle-aged man with misdiagnosed CDC, in order to emphasize the importance of an early and correct diagnosis of this rare disease.

## 2. Case Presentation

A 41-year-old male, heavy smoker, known to have recurrent urinary stones, presented with left flank pain and one episode of gross hematuria. Upon presentation, he was afebrile and chills were not reported.

Physical examination revealed a soft nontender abdomen with left costovertebral angle tenderness and no palpable lymph nodes.

Blood tests including chemistry (complete blood count, electrolytes, blood urea nitrogen, creatinine blood level, and hepatic enzymes) were normal but urinalysis showed 23 White Blood Cells (WBC) and 38 Red Blood Cells (RBC) per high-power microscopic field in urinary sediment.

**Figure 1:**
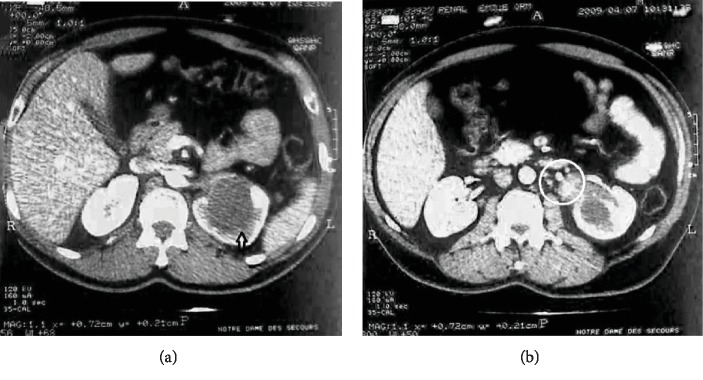
CT scan of the patient showing a 5 cm solid mass of the left renal pelvis (a, arrow) and multiple infracentimetric perihilar lymph nodes (b, circle).

Suspicious urinary cytology warranted further investigations. Therefore, computed tomography (CT) urography scan revealed the presence of a 5 cm left renal pelvis mass and multiple infracentimetric perihilar lymph nodes (Figure 1). Subsequently, laparoscopic left nephroureterectomy with perihilar lymph node dissection was performed a few days later.

**Figure 2:**
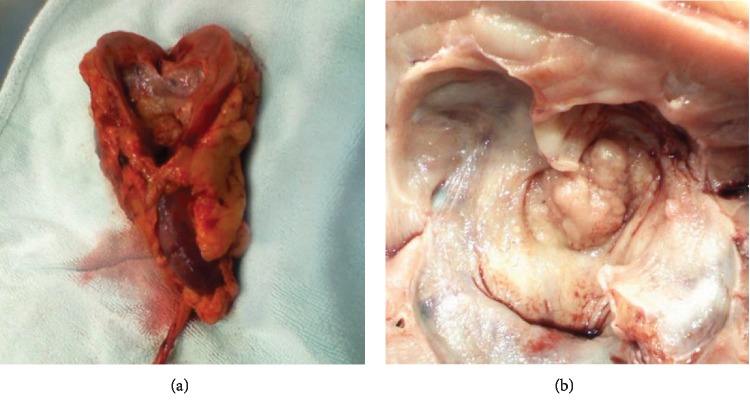
Gross appearance of the left kidney and ureter showing the infiltration of the perihilar fat (a) by a vegetative tumor of the renal pelvis measuring 3 × 2.5 cm and invading the adjacent renal parenchyma (b).

Gross examination revealed a 3 × 2.5 cm vegetative tumor of the renal pelvis that infiltrates the adjacent renal parenchyma and the perihilar fat (Figure 2). Microscopic examination showed a high-grade transitional papillary carcinoma of the renal pelvic lumen infiltrating the muscular layer of the renal pelvis, the adjacent renal parenchyma, and the perihilar fat with the presence of multiple tumoral emboli in the adjacent venous structures. The presence of urothelial carcinoma in situ (CIS) was also noted in the mucosa. Four of the six dissected lymph nodes showed disease metastasis.

The patient had adjuvant chemotherapy based on cisplatin-gemcitabine and was on a surveillance protocol (CT and urinary cytology) for two years with no signs of relapse.

**Figure 3:**
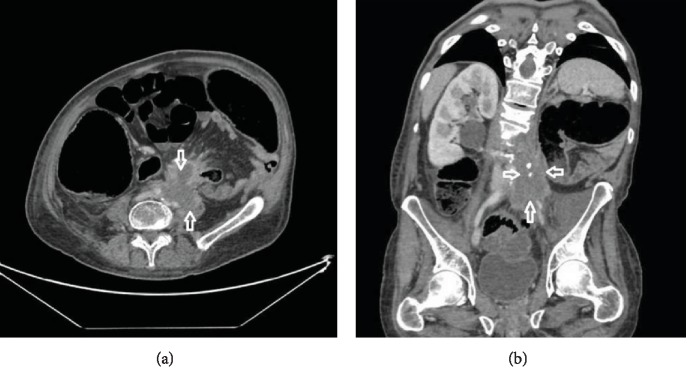
Abdominal CT scan showing a left lateroaortic mass (a) spanning over 12 cm and infiltrating the left psoas and iliac muscles, as well as the left iliac vessels (b).

**Figure 4:**
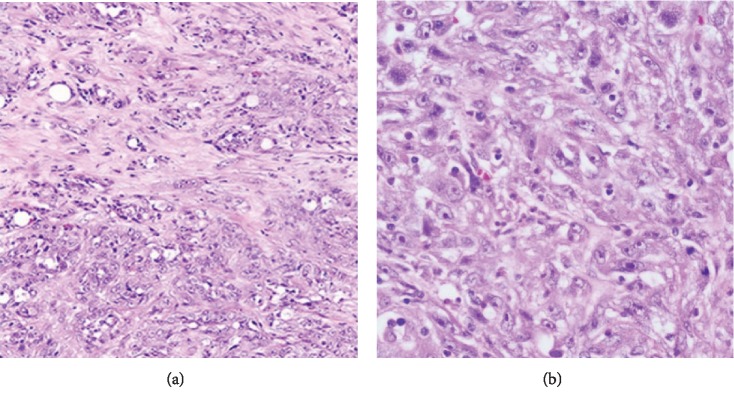
Microscopic appearance of the tumor showing (a) complex, infiltrative, and poorly circumscribed cells with some cords and tubules (×10) and (b) carcinomatous proliferation formed by polygonal cells with eosinophilic and dense cytoplasm and an irregular hyperchromatic nucleus with multiple nucleoli and occasional presence of clear cells (×40).

After that, he was lost of view for one year and returned back again for periumbilical abdominal pain and a recent history of weight loss. Physical examination and laboratory tests were unremarkable. CT scan showed a left paraaortic mass infiltrating the left psoas muscle over a length of 12 cm (Figure 3). Biopsy of this mass revealed carcinomatous proliferation formed by polygonal cells with eosinophilic and dense cytoplasm containing irregular hyperchromatic nucleus with multiple nucleoli. Occasional clear cells were also seen (Figure 4). These findings required further investigations to confirm the origin of the disease. This is why immunohistochemical profiling was done and showed diffuse and strong positivity of tumoral cells for anti-CK7 and anti-HMWCK while having negative results with CD10 and CD117 stains. Based on these findings, the diagnosis of BDC was maintained, rejecting the initial diagnosis of TCC. The patient was subsequently restarted on a cisplatin-gemcitabine-based chemotherapy regimen.

## 3. Discussion

Collecting duct carcinoma (CDC) was first described in 1976 by Mancilla-Jimenez et al. [4] as atypical hyperplastic changes of the epithelium adjacent to the collecting ducts. Fleming and Lewi established diagnostic criteria for this special subtype of renal cell carcinoma (RCC) [3] until it was recognized by the World Health Organization (WHO) in 1998 as a new entity [1].

Till 2013, almost 200 cases have been reported in the literature [5] with less than 5 cases reported later [1, 5]. An increased prevalence of BDC was reported in patients with renal failure and nephrolithiasis and on hemodialysis [6], aligning well with our patient's history of recurrent urinary stones.

Symptoms at presentation may resemble RCC in 70% of the cases (gross hematuria, weakness, flank mass or pain, and weight loss). This fact should always be kept in mind since it contributed to our initial misdiagnosis; our patient mostly presented nonspecific symptoms. The rest may be atypical (acute renal failure, metastatic lesions to bones or meninges, and lymphadenopathy) [5–7]. Common metastasis sites include the lungs, bones (as osteoblastic lesions), liver, and adrenal glands [3, 6]. Metastasis or paraneoplastic symptoms are present in 40% of cases at presentation [1]. One asymptomatic case, diagnosed incidentally as a heterogeneous abdominal mass on ultrasonography (US), was described by Kierstan et al. [6].

Laboratory studies are usually normal, though microscopic hematuria and mild anemia were reported in some cases [2, 5, 6]. Alpha-fetoprotein (AFP), cancer embryonal antigen (CEA), and urine cytology might be positive [6] and, in some cases, such as ours, an important clue for a definite diagnosis.

Imaging findings are nonspecific. Computed tomography commonly shows heterogeneous enhancement of a medullary mass with cystic components, poorly defined contour, and extension to the renal pelvis. Infradiaphragmatic thrombus of the inferior vena cava and multiple regional lymphadenopathies were described as signs of locally advanced disease [5]. In addition to the collecting duct mass, our patient showed multiple infracentimetric perihilar lymph nodes, hence considered a locally advanced disease. Heterogeneous masses, irregular borders, variable echoes, and blood flow signals were described on ultrasonography [2, 6]. MRI reveals an isointense image on T1, iso- or hypointense image on T2, and lower enhancement compared to the normal cortex and medulla [4].

The diagnosis is made on pathology, but it can be difficult since it has many common features with other tumors like urothelial papillary carcinoma (our case). However, the latter can be differentiated from CDC by its positive expression of p63, GATA3, and Uroplakin II, while negative for PAX8 [8]. Macroscopically, the tumor looks like a grey to white mass with cyst-like formations resulting from distention of collecting tubules. Invasion of the renal sinus or the cortex, thrombus of the vena cava, enlarged lymph nodes, arterial embolization, and perirenal tissue necrosis are described. Microscopically, the BDC, as seen also in our case, is described as round to polygonal cells with typical knob-like widened portions (cobblestones), acidophilic cytoplasm, and rarely hyperchromatic nuclei arranged in a tubular glandular pattern with multiple atypical mitoses, anaplastic giant cells, chronic interstitial nephritis, areas of hemorrhage, and necrosis [7]. Synchronous carcinoma in situ or dysplastic lesions are typically seen in the adjacent tubules. Immunohistological studies are positive for cytokeratin cocktail, high-molecular weight cytokeratin, pancytokeratin (AE1/AE3) [5, 6], EMA, and Vimentin [2] but negative for CD117 [5]. CD10 and CK7 status is variable [2, 5, 6]. This immunohistological profiling was the tool we used to determine the origin of our paraaortic mass, beyond any reasonable doubt.

Genetically, the disease is associated with deletion of chromosome 1q or loss of chromosomes 1, 6, 8, 11, 18, and 21 without sufficient data on the molecular mechanisms [6].

Detecting the disease at an early stage is the only favorable prognostic factor [2]. In fact, survival in operated patients with low-grade BDC was 5 times more than in those with high-grade disease [2]. The median overall survival is 7.6 months [3], and more than half of the patients die within 2 years form diagnosis [1, 7] due to the dissemination of the disease [7]. Independent factors associated to disease-specific mortality are the following: American Society of Anesthesiologists (ASA) score 3 and 4, tumor size > 7 cm, stage M1, Fuhrman grade 3 and 4 (despite Vancouver conference's suggestion of not assigning a CDC grade, Ciszewski et al. graded patients based on the Fuhrman scale since they examined them before publication of conference recommendations), and lymphovascular invasion [3].

No definitive treatment is established so far [1]. The treatment of choice is radical nephrectomy due to the central location of the disease and the tendency to invade the collecting system [1]. Surgical treatment offers the longest survival if performed when the disease is still localized in the kidney [3]. It was also associated to adjuvant chemotherapy; knowing that both the collecting duct and the urothelial cells originate from divisions of the mesonephric (Wolff) duct, and based on morphologic, antigenic, and cytogenetic similarities between CDC and urothelial carcinoma (UC), the use of similar chemotherapy regimens has been practiced with promising results for future treatment consideration [9]. Identical to our case, Orsola et al. suggested an association between urothelial carcinoma and CDC and they presented cases where one tumor preceded the other [9]. Our case was considered a diagnostic error more than an evolution of the initial tumor because the previous pathology was not reviewed again. However, the fact that these tumors have the same embryologic origin can highlight the hypothesis that transitional urothelial carcinoma could eventually develop a second type of tumor such as the CDC.

Adjuvant chemotherapy using gemcitabine+cisplatin/carboplatin made 26% remission rate [1], which was also our informed and optimal treatment of choice, following the radical nephrectomy. Treatment with tyrosine kinase inhibitors (sorafenib, sunitinib, or temsirolimus) is promising in metastatic disease but needs more investigations [5]. Percutaneous biopsy in metastatic disease might be beneficial to guide the management of the advanced disease [3]. Radiotherapy has no place in BDC [6].

Mishra et al. in 2016 treated a locally advanced disease with adjuvant chemotherapy with no disease relapse at 10 months [5]. Li et al. reported the case of a patient with T1aN0M0 disease who rejected chemotherapy when informed of possible side effects but remained disease-free 4 years after surgery [2].

## 4. Conclusion

BDC remains one of the rare aggressive subtypes of RCC, having a multitude of initial clinical presentations and an unfavorable prognosis. In this patient, CDC was masquerading as a transitional cell carcinoma that should always be kept in mind as a possible presentation. Corresponding early imaging and histopathology exams are primordial for a correct diagnosis and thus a better prognosis.
